# Supplementary material for: Fishery catch is affected by geographic expansion, fishing down food webs and climate change in Aotearoa, New Zealand
Source: PeerJ. 2023 Sep 21;11:e16070. doi: 10.7717/peerj.16070 (PMC10518166; doi:10.7717/peerj.16070)
Supplement: Supplemental Information 3 — The species and higher taxonomic groups recorded in the full Sea Around Us New Zealand EEZ catch dataset, from 1950–2019, that were excluded from analyses in the present study. Listed is the species/group name, as well as its taxonomic classification. [file peerj-11-16070-s003.docx]

| **Species and Higher Taxonomic Groups** | **Taxonomic Classification** |
| --- | --- |
| Bivalvia | Class |
| Echinoidea | Class |
| Elasmobranchii | Class |
| Gastropoda | Class |
| Scyphozoa | Class |
| Berycidae | Family |
| Bothidae | Family |
| Bramidae | Family |
| Callorhinchidae | Family |
| Carangidae | Family |
| Carcharhinidae | Family |
| Centriscidae | Family |
| Centrolophidae | Family |
| Clupeidae | Family |
| Gadidae | Family |
| Gempylidae | Family |
| Istiophoridae | Family |
| Kyphosidae | Family |
| Labridae | Family |
| Lamnidae | Family |
| Latridae | Family |
| Lithodidae | Family |
| Macrouridae | Family |
| Merlucciidae | Family |
| Monacanthidae | Family |
| Moridae | Family |
| Mugilidae | Family |
| Mullidae | Family |
| Myctophidae | Family |
| Myliobatidae | Family |
| Mytilidae | Family |
| Nephropidae | Family |
| Nototheniidae | Family |
| Ommastrephidae | Family |
| Oreosomatidae | Family |
| Palinuridae | Family |
| Pectinidae | Family |
| Pinguipedidae | Family |
| Portunidae | Family |
| Rajidae | Family |
| Scombridae | Family |
| Scorpaenidae | Family |
| Scyliorhinidae | Family |
| Serranidae | Family |
| Solenoceridae | Family |
| Sparidae | Family |
| Sphyrnidae | Family |
| Squalidae | Family |
| Tetraodontidae | Family |
| Trachichthyidae | Family |
| Triakidae | Family |
| Triglidae | Family |
| Veneridae | Family |
| Xiphiidae | Family |
| Zeidae | Family |
| Alopias | Genus |
| Benthodesmus | Genus |
| Beryx | Genus |
| Coryphaena | Genus |
| Decapterus | Genus |
| Dosinia | Genus |
| Epigonus | Genus |
| Epinephelus | Genus |
| Etmopterus | Genus |
| Haliotis | Genus |
| Hydrolagus | Genus |
| Isurus | Genus |
| Lampris | Genus |
| Nemadactylus | Genus |
| Polyprion | Genus |
| Pterygotrigla | Genus |
| Saccostrea | Genus |
| Seriola | Genus |
| Seriolella | Genus |
| Sphyraena | Genus |
| Sphyrna | Genus |
| Squalus | Genus |
| Trachurus | Genus |
| Trichiurus | Genus |
| Batoidea | Infraclass |
| Anguilliformes | Order |
| Carcharhiniformes | Order |
| Chimaeriformes | Order |
| Clupeiformes | Order |
| Decapoda | Order |
| Gadiformes | Order |
| Lamniformes | Order |
| Perciformes | Order |
| Pleuronectiformes | Order |
| Rajiformes | Order |
| Scorpaeniformes | Order |
| Squaliformes | Order |
| Teuthida | Order |
| Chondrichthyes | Parvphylum |
| Mollusca | Phylum |
| *Allocyttus niger* | Species |
| *Australostichopus mollis* | Species |
| *Centroselachus crepidater* | Species |
| *Cephaloscyllium isabellum* | Species |
| *Chlamys delicatula* | Species |
| *Crassostrea gigas* | Species |
| *Deania calcea* | Species |
| *Emmelichthys nitidus nitidus* | Species |
| *Haliporoides sibogae* | Species |
| *Hoplostethus mediterraneus mediterraneus* | Species |
| *Hyporhamphus ihi* | Species |
| *Jacquinotia edwardsi* | Species |
| *Jasus edwardsii* | Species |
| *Metanephrops challengeri* | Species |
| *Nemadactylus macropterus* | Species |
| *Nototodarus sloanii* | Species |
| *Odax pullus* | Species |
| *Paphies australis* | Species |
| *Pecten novaezelandiae* | Species |
| *Perna canaliculus* | Species |
| *Polyprion oxygeneios* | Species |
| *Pseudocaranx dentex* | Species |
| *Pseudocyttus maculatus* | Species |
| *Pseudopentaceros richardsoni* | Species |
| *Rexea solandri* | Species |
| *Trachurus declivis* | Species |
| *Zeus faber* | Species |
| Dendrobranchiata | Suborder |
| Pleuronectoidei | Suborder |
| Marine finfishes not identified | Unidentified |
| Marine fishes not identified | Unidentified |
| Marine groundfishes not identified | Unidentified |
| Marine pelagic fishes not identified | Unidentified |
| Miscellaneous aquatic invertebrates | Unidentified |
| Miscellaneous marine crustaceans | Unidentified |
