# Supplementary material for: Fishery catch is affected by geographic expansion, fishing down food webs and climate change in Aotearoa, New Zealand
Source: PeerJ. 2023 Sep 21;11:e16070. doi: 10.7717/peerj.16070 (PMC10518166; doi:10.7717/peerj.16070)
Supplement: Supplemental Information 4 — The gear used, from 1950–2019, within New Zealand’s EEZ fisheries catch data, as reported by the Sea Around Us. Right: The broader fisheries gear groupings used in analyses for all listed gear within the fisheries catch data. [file peerj-11-16070-s004.docx]

| **Sea Around Us gear classification** | **Present study gear grouping** |
| --- | --- |
| Small scale gillnets | Small scale |
| Small scale seine nets | Small scale |
| Recreational fishing gear | Small scale |
| Subsistence fishing gear | Small scale |
| Small scale longline | Small scale |
| Artisanal fishing gear | Small scale |
| Small scale hand lines | Small scale |
| Small scale pots or traps | Small scale |
| Small scale purse seine | Small scale |
| Small scale lines | Small scale |
| Small scale other nets | Small scale |
| Longline | Longline |
| Unknown class | Unknown |
| Bottom trawl | Bottom trawl |
| Gillnet | Gillnet |
| Hand lines | Handlines |
| Pots or traps | Pots or traps |
| Purse seine | Purse seine |
| Mixed gear | Mixed gear |
| Other | Other |
| Other nets | Other |
| Pelagic trawl | Pelagic trawl |
| Pole and line | Pole and line |
