# Supplementary material for: Fishery catch is affected by geographic expansion, fishing down food webs and climate change in Aotearoa, New Zealand
Source: PeerJ. 2023 Sep 21;11:e16070. doi: 10.7717/peerj.16070 (PMC10518166; doi:10.7717/peerj.16070)
Supplement: Supplemental Information 5 — The list of study species recorded as catch between the distinct gear groups. Note: Several species are captured in more than one gear. [file peerj-11-16070-s005.docx]

| **Fishing gear** | **Species** |
| --- | --- |
| Bottom trawl | *Aldrichetta forsteri* |
| Bottom trawl | *Alopias superciliosus* |
| Bottom trawl | *Alopias vulpinus* |
| Bottom trawl | *Antimora rostrata* |
| Bottom trawl | *Argentina elongata* |
| Bottom trawl | *Beryx splendens* |
| Bottom trawl | *Brama brama* |
| Bottom trawl | *Callorhinchus milii* |
| Bottom trawl | *Capromimus abbreviatus* |
| Bottom trawl | *Carcharhinus brachyurus* |
| Bottom trawl | *Carcharodon carcharias* |
| Bottom trawl | *Centriscops humerosus* |
| Bottom trawl | *Centroberyx affinis* |
| Bottom trawl | *Centrolophus niger* |
| Bottom trawl | *Centrophorus squamosus* |
| Bottom trawl | *Centroselachus crepidater* |
| Bottom trawl | *Cetorhinus maximus* |
| Bottom trawl | *Chelidonichthys kumu* |
| Bottom trawl | *Coryphaena hippurus* |
| Bottom trawl | *Cyttus novaezealandiae* |
| Bottom trawl | *Cyttus traversi* |
| Bottom trawl | *Dalatias licha* |
| Bottom trawl | *Diastobranchus capensis* |
| Bottom trawl | *Dissostichus eleginoides* |
| Bottom trawl | *Epigonus telescopus* |
| Bottom trawl | *Eptatretus cirrhatus* |
| Bottom trawl | *Galeocerdo cuvier* |
| Bottom trawl | *Galeorhinus galeus* |
| Bottom trawl | *Genyagnus monopterygius* |
| Bottom trawl | *Genypterus blacodes* |
| Bottom trawl | *Girella tricuspidata* |
| Bottom trawl | *Halargyreus johnsonii* |
| Bottom trawl | *Harriotta raleighana* |
| Bottom trawl | *Hoplostethus atlanticus* |
| Bottom trawl | *Hydrolagus novaezealandiae* |
| Bottom trawl | *Hyperoglyphe antarctica* |
| Bottom trawl | *Isurus oxyrinchus* |
| Bottom trawl | *Kathetostoma giganteum* |
| Bottom trawl | *Lamna nasus* |
| Bottom trawl | *Lampris guttatus* |
| Bottom trawl | *Latris lineata* |
| Bottom trawl | *Lepidocybium flavobrunneum* |
| Bottom trawl | *Lepidopus caudatus* |
| Bottom trawl | *Lepidorhynchus denticulatus* |
| Bottom trawl | *Macrourus carinatus* |
| Bottom trawl | *Macruronus novaezelandiae* |
| Bottom trawl | *Magnisudis prionosa* |
| Bottom trawl | *Merluccius australis* |
| Bottom trawl | *Meuschenia scaber* |
| Bottom trawl | *Micromesistius australis* |
| Bottom trawl | *Mola mola* |
| Bottom trawl | *Mora moro* |
| Bottom trawl | *Mugil cephalus* |
| Bottom trawl | *Mustelus lenticulatus* |
| Bottom trawl | *Neocyttus rhomboidalis* |
| Bottom trawl | *Notopogon lilliei* |
| Bottom trawl | *Notorynchus cepedianus* |
| Bottom trawl | *Odontaspis ferox* |
| Bottom trawl | *Pagrus auratus* |
| Bottom trawl | *Parapercis colias* |
| Bottom trawl | *Paratrachichthys trailli* |
| Bottom trawl | *Paristiopterus labiosus* |
| Bottom trawl | *Pentaceros decacanthus* |
| Bottom trawl | *Plagiogeneion rubiginosum* |
| Bottom trawl | *Prionace glauca* |
| Bottom trawl | *Pseudophycis bachus* |
| Bottom trawl | *Regalecus glesne* |
| Bottom trawl | *Ruvettus pretiosus* |
| Bottom trawl | *Sardinops sagax* |
| Bottom trawl | *Scomber australasicus* |
| Bottom trawl | *Scorpis violacea* |
| Bottom trawl | *Seriola lalandi* |
| Bottom trawl | *Seriolella brama* |
| Bottom trawl | *Seriolella caerulea* |
| Bottom trawl | *Seriolella punctata* |
| Bottom trawl | *Sphoeroides pachygaster* |
| Bottom trawl | *Sphyrna zygaena* |
| Bottom trawl | *Squalus acanthias* |
| Bottom trawl | *Squalus mitsukurii* |
| Bottom trawl | *Thyrsites atun* |
| Bottom trawl | *Trachipterus trachypterus* |
| Bottom trawl | *Tripterophycis gilchristi* |
| Bottom trawl | *Zenopsis nebulosa* |
| Gillnet | *Callorhinchus milii* |
| Gillnet | *Galeorhinus galeus* |
| Gillnet | *Genyagnus monopterygius* |
| Gillnet | *Genypterus blacodes* |
| Gillnet | *Girella tricuspidata* |
| Gillnet | *Hyperoglyphe antarctica* |
| Gillnet | *Istiompax indica* |
| Gillnet | *Kajikia audax* |
| Gillnet | *Katsuwonus pelamis* |
| Gillnet | *Latris lineata* |
| Gillnet | *Macruronus novaezelandiae* |
| Gillnet | *Mugil cephalus* |
| Gillnet | *Mustelus lenticulatus* |
| Gillnet | *Pseudophycis bachus* |
| Gillnet | *Seriolella brama* |
| Gillnet | *Squalus acanthias* |
| Gillnet | *Thunnus alalunga* |
| Gillnet | *Thunnus albacares* |
| Gillnet | *Thunnus obesus* |
| Gillnet | *Thunnus orientalis* |
| Gillnet | *Xiphias gladius* |
| Hand lines | *Istiompax indica* |
| Hand lines | *Kajikia audax* |
| Hand lines | *Katsuwonus pelamis* |
| Hand lines | *Parapercis colias* |
| Hand lines | *Thunnus alalunga* |
| Hand lines | *Thunnus albacares* |
| Hand lines | *Thunnus maccoyii* |
| Hand lines | *Thunnus obesus* |
| Hand lines | *Xiphias gladius* |
| Longline | *Alopias vulpinus* |
| Longline | *Carcharhinus falciformis* |
| Longline | *Carcharhinus longimanus* |
| Longline | *Chelidonichthys kumu* |
| Longline | *Coryphaena hippurus* |
| Longline | *Galeorhinus galeus* |
| Longline | *Hyperoglyphe antarctica* |
| Longline | *Istiompax indica* |
| Longline | *Isurus oxyrinchus* |
| Longline | *Kajikia audax* |
| Longline | *Katsuwonus pelamis* |
| Longline | *Lamna nasus* |
| Longline | *Pagrus auratus* |
| Longline | *Parapercis colias* |
| Longline | *Prionace glauca* |
| Longline | *Squalus acanthias* |
| Longline | *Thunnus alalunga* |
| Longline | *Thunnus albacares* |
| Longline | *Thunnus maccoyii* |
| Longline | *Thunnus obesus* |
| Longline | *Thunnus orientalis* |
| Longline | *Xiphias gladius* |
| Mixed gear | *Thunnus maccoyii* |
| Other | *Istiompax indica* |
| Other | *Kajikia audax* |
| Other | *Katsuwonus pelamis* |
| Other | *Thunnus alalunga* |
| Other | *Thunnus albacares* |
| Other | *Thunnus obesus* |
| Other | *Thunnus orientalis* |
| Other | *Xiphias gladius* |
| Pelagic trawl | *Aldrichetta forsteri* |
| Pelagic trawl | *Alepisaurus ferox* |
| Pelagic trawl | *Arripis trutta* |
| Pelagic trawl | *Beryx decadactylus* |
| Pelagic trawl | *Beryx splendens* |
| Pelagic trawl | *Brama brama* |
| Pelagic trawl | *Carcharhinus brachyurus* |
| Pelagic trawl | *Carcharhinus galapagensis* |
| Pelagic trawl | *Centroberyx affinis* |
| Pelagic trawl | *Centrolophus niger* |
| Pelagic trawl | *Coryphaena hippurus* |
| Pelagic trawl | *Galeorhinus galeus* |
| Pelagic trawl | *Genyagnus monopterygius* |
| Pelagic trawl | *Girella tricuspidata* |
| Pelagic trawl | *Lamna nasus* |
| Pelagic trawl | *Lampris guttatus* |
| Pelagic trawl | *Latris lineata* |
| Pelagic trawl | *Lepidocybium flavobrunneum* |
| Pelagic trawl | *Macruronus novaezelandiae* |
| Pelagic trawl | *Micromesistius australis* |
| Pelagic trawl | *Mola mola* |
| Pelagic trawl | *Mora moro* |
| Pelagic trawl | *Mugil cephalus* |
| Pelagic trawl | *Pagrus auratus* |
| Pelagic trawl | *Plagiogeneion rubiginosum* |
| Pelagic trawl | *Ruvettus pretiosus* |
| Pelagic trawl | *Seriola lalandi* |
| Pelagic trawl | *Squalus mitsukurii* |
| Pelagic trawl | *Thyrsites atun* |
| Pole and line | *Katsuwonus pelamis* |
| Pole and line | *Thunnus alalunga* |
| Pole and line | *Thunnus albacares* |
| Pole and line | *Thunnus obesus* |
| Pole and line | *Thunnus orientalis* |
| Pots or traps | *Parapercis colias* |
| Purse seine | *Carcharhinus falciformis* |
| Purse seine | *Carcharhinus longimanus* |
| Purse seine | *Coryphaena hippurus* |
| Purse seine | *Istiompax indica* |
| Purse seine | *Kajikia audax* |
| Purse seine | *Katsuwonus pelamis* |
| Purse seine | *Scomber australasicus* |
| Purse seine | *Thunnus alalunga* |
| Purse seine | *Thunnus albacares* |
| Purse seine | *Thunnus maccoyii* |
| Purse seine | *Thunnus obesus* |
| Purse seine | *Thunnus orientalis* |
| Purse seine | *Thyrsites atun* |
| Purse seine | *Xiphias gladius* |
| Small scale | *Aldrichetta forsteri* |
| Small scale | *Allothunnus fallai* |
| Small scale | *Alopias superciliosus* |
| Small scale | *Alopias vulpinus* |
| Small scale | *Antimora rostrata* |
| Small scale | *Argentina elongata* |
| Small scale | *Arripis trutta* |
| Small scale | *Beryx splendens* |
| Small scale | *Brama brama* |
| Small scale | *Callorhinchus milii* |
| Small scale | *Capromimus abbreviatus* |
| Small scale | *Carcharhinus brachyurus* |
| Small scale | *Carcharodon carcharias* |
| Small scale | *Centriscops humerosus* |
| Small scale | *Centroberyx affinis* |
| Small scale | *Centrolophus niger* |
| Small scale | *Centrophorus squamosus* |
| Small scale | *Centroselachus crepidater* |
| Small scale | *Cetorhinus maximus* |
| Small scale | *Chelidonichthys kumu* |
| Small scale | *Coryphaena hippurus* |
| Small scale | *Cyttus novaezealandiae* |
| Small scale | *Cyttus traversi* |
| Small scale | *Dalatias licha* |
| Small scale | *Diastobranchus capensis* |
| Small scale | *Epigonus telescopus* |
| Small scale | *Eptatretus cirrhatus* |
| Small scale | *Galeocerdo cuvier* |
| Small scale | *Galeorhinus galeus* |
| Small scale | *Genyagnus monopterygius* |
| Small scale | *Genypterus blacodes* |
| Small scale | *Girella tricuspidata* |
| Small scale | *Halargyreus johnsonii* |
| Small scale | *Harriotta raleighana* |
| Small scale | *Hoplostethus atlanticus* |
| Small scale | *Hydrolagus novaezealandiae* |
| Small scale | *Hyperoglyphe antarctica* |
| Small scale | *Istiophorus platypterus* |
| Small scale | *Isurus oxyrinchus* |
| Small scale | *Kajikia audax* |
| Small scale | *Kathetostoma giganteum* |
| Small scale | *Katsuwonus pelamis* |
| Small scale | *Lamna nasus* |
| Small scale | *Lampris guttatus* |
| Small scale | *Latris lineata* |
| Small scale | *Lepidocybium flavobrunneum* |
| Small scale | *Lepidopus caudatus* |
| Small scale | *Lepidorhynchus denticulatus* |
| Small scale | *Macrourus carinatus* |
| Small scale | *Macruronus novaezelandiae* |
| Small scale | *Magnisudis prionosa* |
| Small scale | *Makaira mazara* |
| Small scale | *Merluccius australis* |
| Small scale | *Meuschenia scaber* |
| Small scale | *Micromesistius australis* |
| Small scale | *Mola mola* |
| Small scale | *Mora moro* |
| Small scale | *Mugil cephalus* |
| Small scale | *Mustelus lenticulatus* |
| Small scale | *Neocyttus rhomboidalis* |
| Small scale | *Notopogon lilliei* |
| Small scale | *Notorynchus cepedianus* |
| Small scale | *Odontaspis ferox* |
| Small scale | *Pagrus auratus* |
| Small scale | *Parapercis colias* |
| Small scale | *Paratrachichthys trailli* |
| Small scale | *Paristiopterus labiosus* |
| Small scale | *Pentaceros decacanthus* |
| Small scale | *Plagiogeneion rubiginosum* |
| Small scale | *Prionace glauca* |
| Small scale | *Pseudophycis bachus* |
| Small scale | *Regalecus glesne* |
| Small scale | *Ruvettus pretiosus* |
| Small scale | *Sardinops sagax* |
| Small scale | *Scomber australasicus* |
| Small scale | *Scorpis violacea* |
| Small scale | *Seriola lalandi* |
| Small scale | *Seriolella brama* |
| Small scale | *Seriolella caerulea* |
| Small scale | *Seriolella punctata* |
| Small scale | *Sphoeroides pachygaster* |
| Small scale | *Sphyrna zygaena* |
| Small scale | *Squalus acanthias* |
| Small scale | *Squalus mitsukurii* |
| Small scale | *Tetrapturus angustirostris* |
| Small scale | *Thunnus alalunga* |
| Small scale | *Thunnus albacares* |
| Small scale | *Thunnus maccoyii* |
| Small scale | *Thunnus obesus* |
| Small scale | *Thunnus orientalis* |
| Small scale | *Thyrsites atun* |
| Small scale | *Trachipterus trachypterus* |
| Small scale | *Tripterophycis gilchristi* |
| Small scale | *Xiphias gladius* |
| Small scale | *Zenopsis nebulosa* |
| Unknown | *Aldrichetta forsteri* |
| Unknown | *Alopias superciliosus* |
| Unknown | *Alopias vulpinus* |
| Unknown | *Antimora rostrata* |
| Unknown | *Argentina elongata* |
| Unknown | *Arripis trutta* |
| Unknown | *Beryx splendens* |
| Unknown | *Brama brama* |
| Unknown | *Callorhinchus milii* |
| Unknown | *Capromimus abbreviatus* |
| Unknown | *Carcharhinus brachyurus* |
| Unknown | *Carcharodon carcharias* |
| Unknown | *Centriscops humerosus* |
| Unknown | *Centroberyx affinis* |
| Unknown | *Centrolophus niger* |
| Unknown | *Centrophorus squamosus* |
| Unknown | *Centroselachus crepidater* |
| Unknown | *Cetorhinus maximus* |
| Unknown | *Chelidonichthys kumu* |
| Unknown | *Coryphaena hippurus* |
| Unknown | *Cyttus novaezealandiae* |
| Unknown | *Cyttus traversi* |
| Unknown | *Dalatias licha* |
| Unknown | *Diastobranchus capensis* |
| Unknown | *Dissostichus eleginoides* |
| Unknown | *Epigonus telescopus* |
| Unknown | *Eptatretus cirrhatus* |
| Unknown | *Galeocerdo cuvier* |
| Unknown | *Galeorhinus galeus* |
| Unknown | *Genypterus blacodes* |
| Unknown | *Halargyreus johnsonii* |
| Unknown | *Harriotta raleighana* |
| Unknown | *Hoplostethus atlanticus* |
| Unknown | *Hydrolagus novaezealandiae* |
| Unknown | *Isurus oxyrinchus* |
| Unknown | *Kathetostoma giganteum* |
| Unknown | *Lamna nasus* |
| Unknown | *Lampris guttatus* |
| Unknown | *Lepidocybium flavobrunneum* |
| Unknown | *Lepidopus caudatus* |
| Unknown | *Lepidorhynchus denticulatus* |
| Unknown | *Macrourus carinatus* |
| Unknown | *Macruronus novaezelandiae* |
| Unknown | *Magnisudis prionosa* |
| Unknown | *Merluccius australis* |
| Unknown | *Meuschenia scaber* |
| Unknown | *Micromesistius australis* |
| Unknown | *Mola mola* |
| Unknown | *Mora moro* |
| Unknown | *Mustelus lenticulatus* |
| Unknown | *Neocyttus rhomboidalis* |
| Unknown | *Notopogon lilliei* |
| Unknown | *Notorynchus cepedianus* |
| Unknown | *Odontaspis ferox* |
| Unknown | *Pagrus auratus* |
| Unknown | *Paratrachichthys trailli* |
| Unknown | *Paristiopterus labiosus* |
| Unknown | *Pentaceros decacanthus* |
| Unknown | *Plagiogeneion rubiginosum* |
| Unknown | *Prionace glauca* |
| Unknown | *Pseudophycis bachus* |
| Unknown | *Regalecus glesne* |
| Unknown | *Ruvettus pretiosus* |
| Unknown | *Sardinops sagax* |
| Unknown | *Scomber australasicus* |
| Unknown | *Scorpis violacea* |
| Unknown | *Seriola lalandi* |
| Unknown | *Seriolella brama* |
| Unknown | *Seriolella caerulea* |
| Unknown | *Seriolella punctata* |
| Unknown | *Sphoeroides pachygaster* |
| Unknown | *Sphyrna zygaena* |
| Unknown | *Squalus acanthias* |
| Unknown | *Squalus mitsukurii* |
| Unknown | *Thyrsites atun* |
| Unknown | *Trachipterus trachypterus* |
| Unknown | *Tripterophycis gilchristi* |
| Unknown | *Zenopsis nebulosa* |
