# Supplementary material for: Fishery catch is affected by geographic expansion, fishing down food webs and climate change in Aotearoa, New Zealand
Source: PeerJ. 2023 Sep 21;11:e16070. doi: 10.7717/peerj.16070 (PMC10518166; doi:10.7717/peerj.16070)
Supplement: Supplemental Information 6 — Results from simple linear regression and segmented linear regression for the Mean Temperature of the Catch (MTC, °C), the Mean Trophic Level (MTL), and the Fishing-in-Balance Index (FiB) for New Zealand fisheries catch data from FAO catch statistics and the reduced Sea Around Us dataset (n = 42). For each index is listed the time period to which the regression applies, the decadal rate of change of the given index, the Adjusted R2 value, the p-value, as well as the year (± standard error SE) of a segmented regression breakpoint. [file peerj-11-16070-s006.docx]

| **Dataset** | **Index** | **Years** | **Decadal rate of change** | **Adj. *R*^2^** | ***p*-value** | **Breakpoint ± SE** |
| --- | --- | --- | --- | --- | --- | --- |
| FAO (*n*=42) | MTC | 1950-2019 | -1.42 | 0.77 | < 0.01 |  |
|  |  | 1950-1996  1996-2019 | -1.99  0.15 | 0.84 | < 0.01 | 1996 ± 3.1 |
|  | MTL | 1950-2019 | 0.11 | 0.77 | < 0.01 |  |
|  |  | 1950-1997  1997-2019 | 0.15  -0.03 | 0.84 | < 0.01 | 1997 ± 2.9 |
|  | FiB | 1950-2019 | 0.38 | 0.85 | < 0.01 |  |
|  |  | 1950-1999  1999-2019 | 0.52  -0.11 | 0.93 | < 0.01 | 1999 ± 1.9 |
| Reduced Sea Around Us dataset (*n*=42) | MTC | 1950-2019 | -1.42 | 0.77 | < 0.01 |  |
|  |  | 1950-1996  1996-2019 | -1.99  0.15 | 0.84 | < 0.01 | 1996 ± 3.1 |
|  | MTL | 1950-2019 | 0.11 | 0.77 | < 0.01 |  |
|  |  | 1950-1997  1997-2019 | 0.15  -0.03 | 0.84 | < 0.01 | 1997 ± 2.9 |
|  | FiB | 1950-2019 | 0.38 | 0.85 | < 0.01 |  |
|  |  | 1950-1999  1999-2019 | 0.52  -0.11 | 0.93 | < 0.01 | 1999 ± 1.9 |
