# Supplementary material for: Fishery catch is affected by geographic expansion, fishing down food webs and climate change in Aotearoa, New Zealand
Source: PeerJ. 2023 Sep 21;11:e16070. doi: 10.7717/peerj.16070 (PMC10518166; doi:10.7717/peerj.16070)
Supplement: Supplemental Information 7 — Results from simple linear regression and segmented linear regression for the Mean Temperature of the Catch (MTC, °C) of New Zealand fisheries data, separated by species’ milieu and by fishing gear between FAO catch data and the reduced Sea Around Us data (n = 42). The FAO data only analyzed species’ milieu, while the reduced Sea Around Us data analyzed milieu and gear. Included is the time period to which the regression applies to, the decadal rate of change, the Adjusted R2 value, the p-value, as well as the location of a segmented regression breakpoint (± standard error SE). Also listed is each MTC’s Kendall’s τ correlation value (and p-value) with Sea Surface Temperature Anomaly (SSTA) and the full Sea Around Us MTC trend. [file peerj-11-16070-s007.docx]

| **FAO data (*n*=42)** | | | | | | | |
| --- | --- | --- | --- | --- | --- | --- | --- |
| **Milieu** | **Years** | **Decadal rate of change** | **Adj. *R*^2^** | ***p*-value** | **Breakpoint ± SE** | **Kendall’s τ corr. with SSTA (*p*-val)** | **Kendall’s τ corr. with MTC (*p*-val)** |
| Bathydemersal | 1950-2019 | 0.13 | 0.66 | < 0.01 |  | 0.12 (*p*=0.20) | -0.30 (*p*=<0.01) |
|  |  |  |  |  |  |  |  |
| Bathypelagic | 1979-2019 | 0.32 | 0.78 | < 0.01 |  | -0.23 (*p*=0.04) | 0.36 (*p*=<0.01) |
| Benthopelagic | 1950-2019 | -0.71 | 0.83 | < 0.01 |  | -0.20 (*p*=0.02) | 0.53 (*p*=<0.01) |
|  | 1950-1998  1998-2019 | -0.94  0.01 | 0.90 | < 0.01 | 1998 ± 2.70 |  |  |
| Demersal | 1950-2019 | -0.55 | 0.59 | < 0.01 |  | -0.14 (*p*=0.09) | 0.54 (*p*=<0.01) |
|  | 1950-1998  1998-2019 | -0.86  0.52 | 0.72 | < 0.01 | 1998 ± 2.80 |  |  |
| Pelagic-neritic | 1964-2019 | 0.08 | 0.06 | 0.03 |  | -0.09 (*p*=0.35) | -0.09 (*p*=0.32) |
| Pelagic-oceanic | 1953-2019 | 1.68 | 0.17 | < 0.01 |  | 0.05 (*p*=0.59) | 0.09 (*p*=0.38) |
|  | 1953-2009  2009-2019 | 3.12  -11.6 | 0.40 | < 0.01 | 2009 ± 2.20 |  |  |
| **Sea Around Us data (*n*=42)** | | | | | | | |
| **Milieu or gear group** | **Years** | **Decadal rate of change** | **Adj. *R*^2^** | ***p*-value** | **Breakpoint ± SE** | **Kendall’s τ corr. with SSTA (*p*-val)** | **Kendall’s τ corr. with MTC (*p*-val)** |
| Bathydemersal | 1950-2019 | 0.14 | 0.77 | < 0.01 |  | 0.05 (*p*=0.56) | -0.50 (*p*=<0.01) |
|  |  |  |  |  |  |  |  |
| Bathypelagic | 1975-2019 | 0.12 | 0.78 | < 0.01 |  | < 0.01 (*p*=0.98) | -0.38 (*p*=<0.01) |
|  | 1975-1981  1981-2019 | 3.14  -0.04 | 0.82 | < 0.01 | 1981 ± 0.47 |  |  |
| Benthopelagic | 1950-2019 | -0.57 | 0.57 | < 0.01 |  | -0.11 (*p*=0.16) | 0.71 (*p*=<0.01) |
|  | 1950-1982  1982-2019 | -1.33  0.03 | 0.78 | < 0.01 | 1982 ± 2.53 |  |  |
| Demersal | 1950-2019 | -0.44 | 0.50 | < 0.01 |  | -0.05 (*p*=0.57) | 0.58 (*p*=<0.01) |
|  | 1950-1995  1995-2019 | -0.84  0.57 | 0.72 | < 0.01 | 1995 ± 2.24 |  |  |
| Pelagic-neritic | 1950-2019 | -0.01 | < 0.01 | 0.71 |  | 0.06 (*p*=0.50) | 0.23 (*p*=<0.01) |
|  | 1950-1979  1979-2019 | -0.56  0.33 | 0.44 | < 0.01 | 1979 ± 2.58 |  |  |
| Pelagic-oceanic | 1950-2019 | 0.89 | 0.21 | < 0.01 |  | 0.07 (*p*=0.39) | -0.33 (*p*=<0.01) |
| Bottom trawl | 1951-2019 | -1.43 | 0.71 | < 0.01 |  | -0.12 (*p*=0.13) | 0.83 (*p*=<0.01) |
|  | 1951-1987  1987-2019 | -2.90  0.25 | 0.92 | < 0.01 | 1987 ± 1.43 |  |  |
| Gillnet | 1951-2019 | -0.65 | 0.73 | < 0.01 |  | -0.15 (*p*=0.98) | 0.50 (*p*=<0.01) |
| Handline | 1951-2019 | -0.20 | 0.01 | 0.19 |  | 0.04 (*p*=0.61) | 0.31 (*p*=<0.01) |
| Longline | 1950-2019 | -0.40 | 0.11 | < 0.01 |  | 0.03 (*p*=0.73) | 0.12 (*p*=0.15) |
|  | 1950-1970  1970-2019 | -3.40  0.38 | 0.47 | < 0.01 | 1970 ± 2.46 |  |  |
| Other | 1955-2018 | -0.16 | 0.21 | < 0.01 |  | -0.06 (*p*=0.49) | 0.21 (*p*=0.01) |
| Purse seine | 1951-2019 | -0.08 | < 0.01 | 0.74 |  | -0.13 (*p*=0.11) | -0.08 (*p*=0.34) |
| Small scale | 1950-2019 | -1.20 | 0.68 | < 0.01 |  | -0.13 (*p*=0.12) | 0.84 (*p*=<0.01) |
|  | 1950-1987  1987-2019 | -2.35  0.28 | 0.89 | < 0.01 | 1987 ± 1.81 |  |  |
| Unknown | 1951-2019 | -0.84 | 0.30 | < 0.01 |  | 0.01 (*p*=0.93) | 0.52 (*p*=<0.01) |
|  | 1951-1979  1979-2019 | -3.73  0.89 | 0.86 | < 0.01 | 1979 ± 1.18 |  |  |
