# Supplementary figures and images for: Fishery catch is affected by geographic expansion, fishing down food webs and climate change in Aotearoa, New Zealand
Source: PeerJ. 2023 Sep 21;11:e16070. doi: 10.7717/peerj.16070 (PMC10518166; doi:10.7717/peerj.16070)

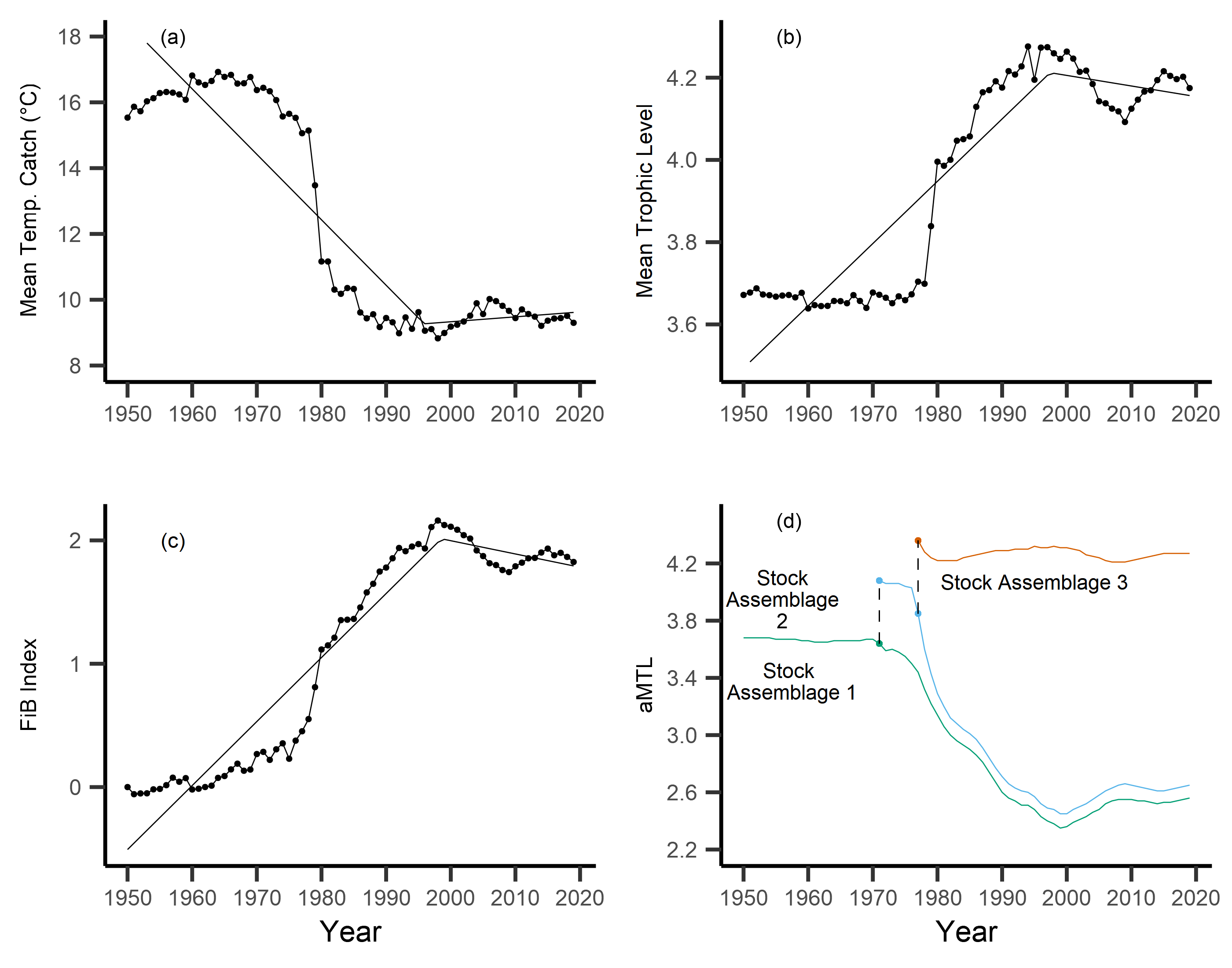

Supplement: Supplemental Information 8 — Trends for the various indices calculated across the New Zealand Exclusive Economic Zone (EEZ) and for FAO fisheries catch data (n = 42) from 1950–2019, including: (a) the mean temperature of the catch (MTC, °C), (b) the mean trophic level (MTL), and (c) the Fishing-in-Balance (FiB) index, including their segmented regression slopes. (d) Results from calculating the adapted Mean Trophic Level (aMTL), including the three identified stock assemblages (green, blue and red lines), as well as the years identified for node expansions (open circles) to include new stock assemblages (dashed lines). The first identified expansion from Stock Assemblage 1 to Stock Assemblage 2 occurred in 1971, while the second expansion from Stock Assemblage 2 to Stock Assemblage 3 occurred in 1977. [file peerj-11-16070-s008.png]

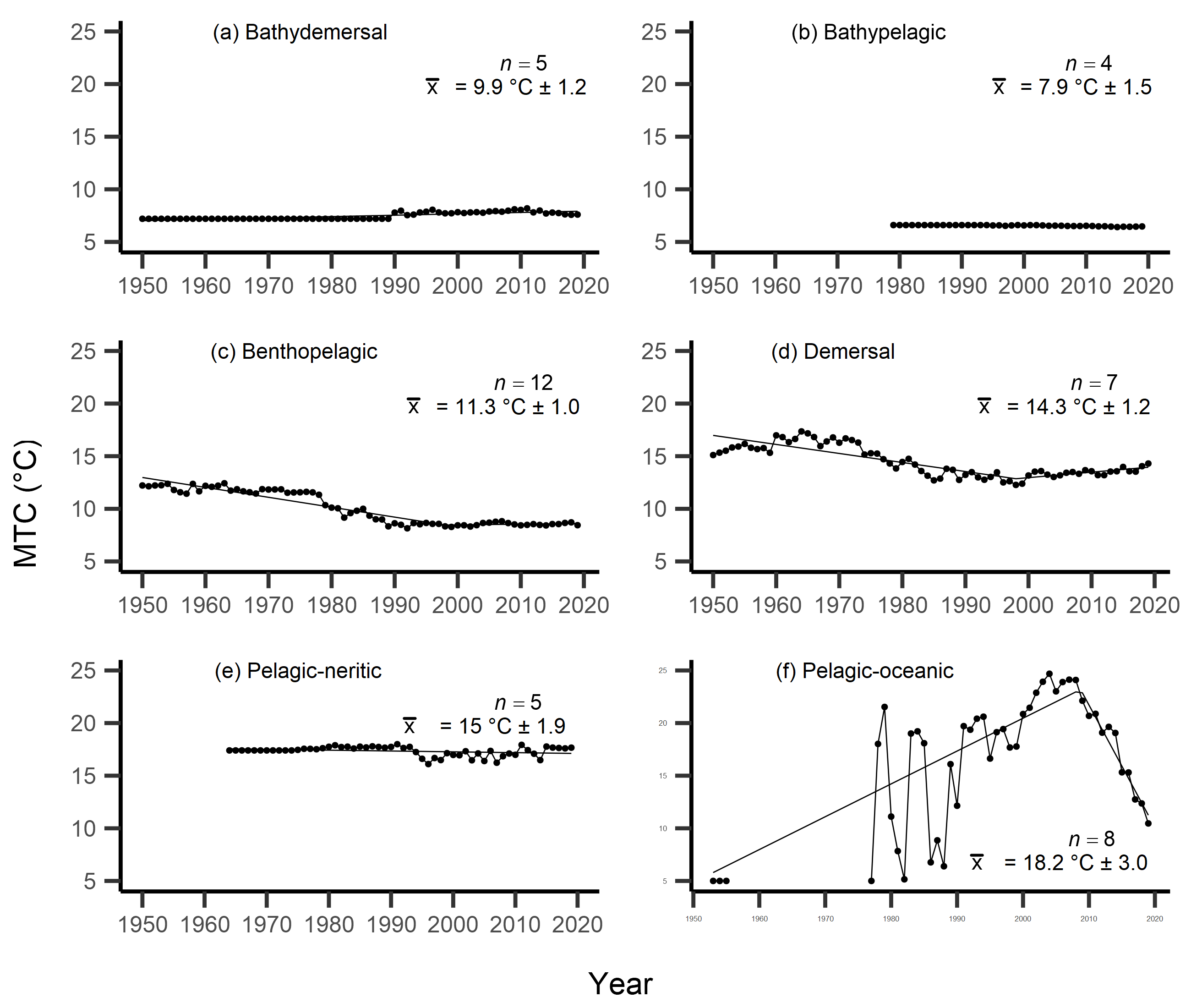

Supplement: Supplemental Information 9 — The mean temperature of the catch (MTC, °C), of New Zealand’s fisheries from 1950–2019, separated by species’ milieu, from FAO catch data (n=42). This includes: (a) bathydemersal, (b) bathypelagic, (c) benthopelagic, (d) demersal, (e) pelagic-neritic, and (f) pelagic-oceanic species. Listed in panels is the number of species included in each milieu (n), as well as the mean temperature preference (°C, ± SE) of all included species. [file peerj-11-16070-s009.png]

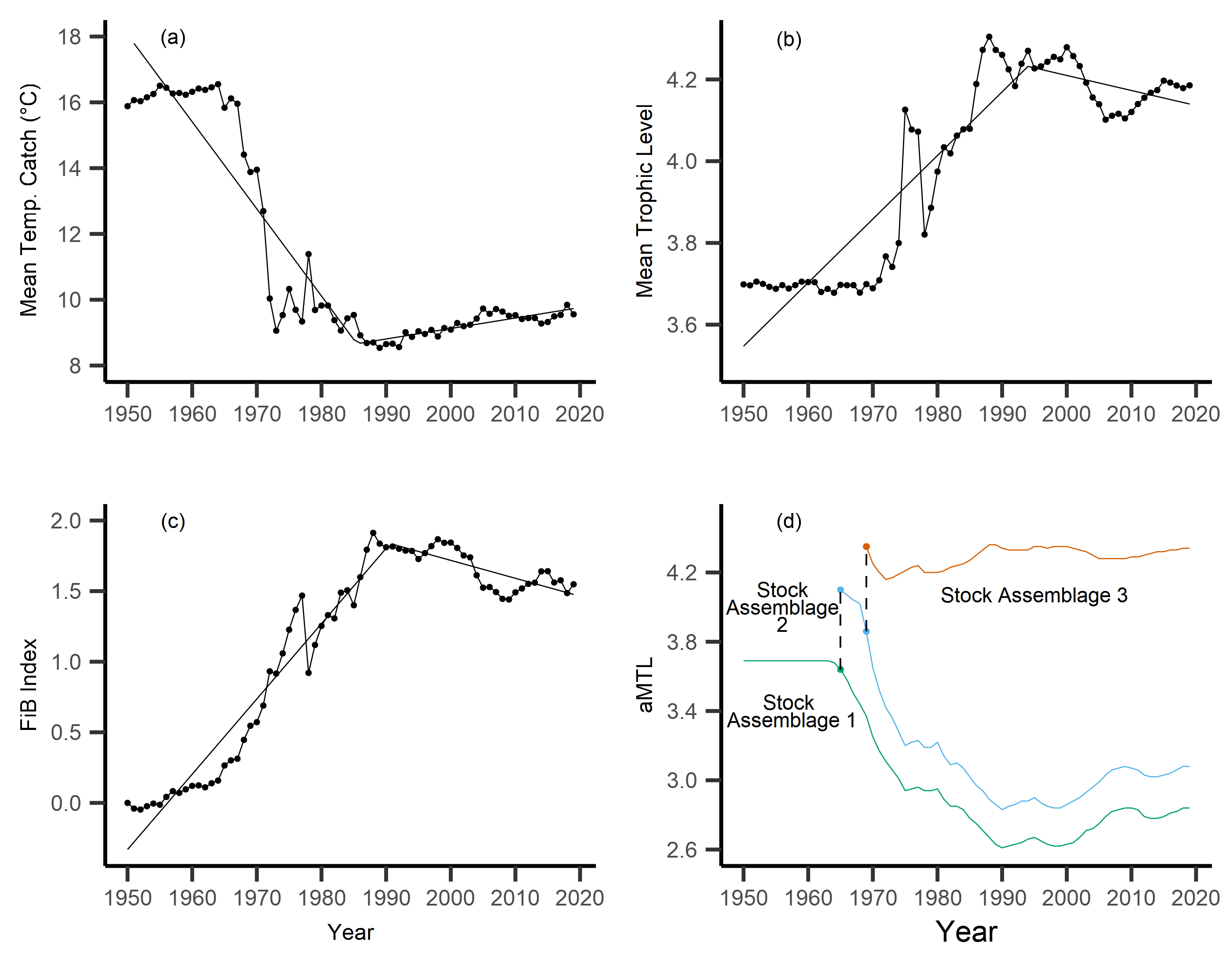

Supplement: Supplemental Information 10 — Trends for the various indices calculated across the New Zealand Exclusive Economic Zone (EEZ) from 1950-2019 using the reduced Sea Around Us (n = 42) dataset, including: (a) the mean temperature of the catch (MTC, °C), (b) the mean trophic level (MTL), and (c) the Fishing-in-Balance (FiB) index, including their segmented regression slopes. (d) Results from calculating the adapted Mean Trophic Level (aMTL), including the three identified stock assemblages (green, blue and red lines), as well as the years identified for node expansions (open circles) to include new stock assemblages (dashed lines). The first identified expansion from Stock Assemblage 1 to Stock Assemblage 2 occurred in 1965, while the second expansion from Stock Assemblage 2 to Stock Assemblage 3 occurred in 1969. [file peerj-11-16070-s010.png]

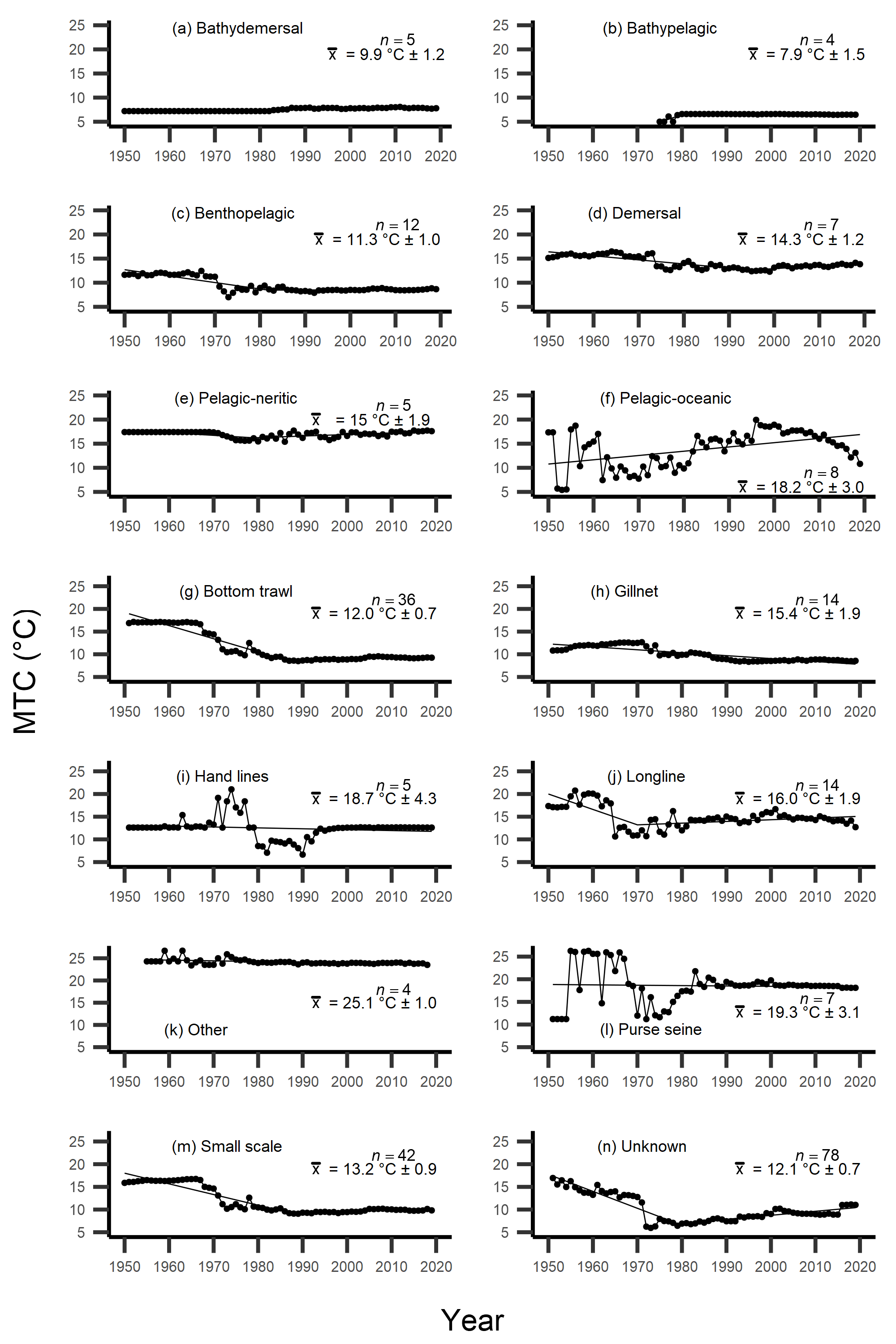

Supplement: Supplemental Information 11 — The mean temperature of the catch (MTC, °C), of New Zealand’s fisheries from 1950–2019, separated by species’ milieu, from the reduced (n = 42) Sea Around Us dataset. This includes: (a) bathydemersal, (b) bathypelagic, (c) benthopelagic, (d) demersal, (e) pelagic-neritic, and (f) pelagic-oceanic species, plus (g) bottom trawl, (h) gillnet, (i) handline, (j) longline, (k) other, (l) purse seine, (m) small scale, and (n) unknown fishing gear groups. Listed in panels is the number of species included in each group (n), as well as the mean temperature preference (°C, ± SE) of all included species. [file peerj-11-16070-s011.png]
